# Effect of silibinin on the expression of MMP2, MMP3, MMP9 and TIMP2 in kidney and lung after hepatic ischemia/reperfusion injury in an experimental rat model

**DOI:** 10.1590/ACB360904

**Published:** 2021-11-08

**Authors:** Vasileios Kollaras, Georgia Valsami, Maria Lambropoulou, Ourania Konstandi, Nikolaos Kostomistsopoulos, Emmanouil Pikoulis, Constantinos Simopoulos, Alexandra Tsaroucha

**Affiliations:** 1 MSc. Postgraduate Program in Hepatobiliary/Pancreatic Surgery - Faculty of Medicine - Democritus University of Thrace - Dragana, Greece.; 2 PhD. School of Health Sciences - Department of Pharmacy - National and Kapodistrian University of Athens, Greece.; 3 PhD. Laboratory of Histology-Embryology - Faculty of Medicine - Democritus University of Thrace - Dragana, Greece.; 4 PhD. Faculty of Cell Biology and Biophysics - Department of Biology - School of Science - National and Kapodistrian University of Athens, Greece.; 5 DVM, PhD. Department of Experimental Surgery - Bioresearch Foundation of the Academy of Athens - Athens, Greece.; 6 PhD. 3rd Department of Surgery - School of Medicine - National and Kapodistrian University of Athens - Attikon Hospital, Greece.; 7 PhD. 2nd Department of Surgery - Faculty of Medicine - Democritus University of Thrace - Alexandroupolis, Greece.; 8 PhD. Laboratory of Experimental Surgery and Surgical Research - Faculty of Medicine - Democritus University of Thrace - Dragana, Greece.

**Keywords:** Silybin, Reperfusion Injury, Immunohistochemistry, Liver, Rats

## Abstract

**Purpose:**

The protective effect of silibinin on kidney and lung parenchyma during hepatic ischemia/reperfusion injury (IRI) is explored.

**Methods:**

Sixty-three Wistar rats were separated into three groups: sham; control (45 min IRI); and silibinin (200 μL silibinin administration after 45 min of ischemia and before reperfusion). Immunohistochemistry and real-time quantitative reverse transcription polymerase chain reaction (qRT-PCR) were used to evaluate the expression levels of MMP2, MMP3, MMP9, and TIMP2 on kidney and lung.

**Results:**

Comparing sham *vs*. control groups, confirmed that hepatic IRI increased both renal and lung MMP2, MMP3, MMP9 and TIMP2 expressions starting at 180 min (p<0.001). Comparison of the control *vs*. silibinin groups showed a statistically significant decrease in the expression levels of MMP2, MMP3, and MMP9 and increase of TIMP2 in kidney and lung parenchyma. The starting point of this decrease was at 120 min after reperfusion, both for kidney and lung parameters, and it was statistically significant at 240 min (p<0.001) for kidney, while silibinin showed a peak of lung protection at 180 min after hepatic reperfusion (p<0.001).

**Conclusions:**

Hepatic IRI causes distant kidney and lung damage, while a statistically significant protective action, both on kidney and lung parenchyma, is conveyed by the intravenous administration of silibinin.

## Introduction

In liver surgery, clinical situations exist in which periods of ischemia are required, such as during trauma, removal of liver tumors, vascular reconstruction, and transplantation^1^^-^ 3. One of the most common, time honored, blood inflow control manipulations is the Pringle maneuver, which is performed by clamping the hepatic pedicle, thereby occluding both portal vein and hepatic artery^4^. This process results in severe liver injury and disfunction^5^^-^ 9, making ischemia reperfusion injury a major cause of morbidity and mortality in liver resection and liver transplantation surgery^10^^,^ 11. Ischemia reperfusion injury, aside from the hepatic damage, also affects other remote organs such as the kidneys, the lungs, the myocardium, the adrenal glands, and the small intestine^12^.

The etiology of acute kidney injury (AKI) is thought to be multifactorial and is usually attributed to renal ischemia due to hemodynamic instability in the perioperative period^13^. In rodents, hepatic ischemia/reperfusion injury (IRI) has been shown to cause AKI^14^^,^ 15, although it is not clear at what stage of the IRI process AKI occurs. There is emerging evidence that suggests multiple molecular mechanisms in the pathophysiology of AKI associated with liver IRI. The first step seems to be portal hypertension due to portal pedicle occlusion. This induces splanchnic vasodilation followed by intrarenal vasoconstriction^16^^-^ 18, that causes activation of the renin-angiotensin-aldosterone axis^12^^,^ 19. This results in glomerular filtration rate reduction, disturbances in the excretion of sodium and water, acute tubular necrosis and renal failure^12^^,^ 16 ^,^ 20. Kidney injury worsens by the activation of pro-inflammatory cytokines (TNF-a, IL-6, IL-1), that drive renal parenchyma to appear significant inflammatory response.

Acute lung injury (ALI) induced by hepatic IRI also involves numerous risk factors^21^^,^ 22. One of the main mechanisms involved is excessive production of reactive oxygen species (ROS) after liver IRI^23^^-^ 25. Excessive production of ROS causes ALI by oxidative stress^26^^,^ 27, inflammatory responses and apoptosis^28^^-^ 30. ROS is related to the injury of the alveolar-capillary membrane and consequent transudation, that contributes to acute respiratory distress syndrome (ARDS)^25^. Significant morphological changes can be observed, such as perivascular edema and intravascular platelet aggregation revealing the deleterious effects of liver IRI on lungs^23^.

In this research protocol, the grade of hepatic IRI induced AKI and ALI is assessed by the expression of matrix metalloproteinases (MMPs), namely MMP2, MMP3, MMP9 and one of their inhibitors, TIMP2, that belong to a family of zinc dependent endopeptidases, capable of degrading extracellular matrix^31^. MMPs are associated with the breakdown of the glomerular basement membrane, renal scarring and fibrosis during the progression of kidney disease^32^^-^ 34. MMPs and their endogenous tissue inhibitors are involved in the pathogenesis of many lung diseases^35^^,^ 36 through extracellular matrix degradation and modulation of inflammation and fibrosis^37^.

Given that hepatic IRI induced AKI and ALI are common complications^12^, effective and preventive strategies are needed. Silibinin is a natural product that forms the major constituent of milk thistle seeds extract^38^ and it has promising hepatoprotective effects owing to its antioxidant and anti-inflammatory properties^39^^-^ 42. *In-vitro* studies have demonstrated anti-cancer effects against several types of cancer^38^. At the molecular level, silibinin decreases inflammatory responses through inhibition of Nrf2 and Nf-kB signaling and suppressing the production of inflammatory cytokines, especially TNF-α^40^^,^ 41.

However, silibinin’s extremely low water-solubility and the extensive first pass metabolism from liver limit its oral administration, while also prevent injectable administration^43^^,^ 44. Fortunately, the recently developed and *in-vitro* and *in-vivo* evaluated water-soluble lyophilized product of silibinin with hydroxypropyl-β-cyclodextrin (SLB-HP-β-CD) was proven to be 10 times more bioavailable than pure silibinin^43^^,^ 44. Furthermore, our previous observations^45^^-^ 47 revealed that silibinin, when administered *iv* in the form of SLB-HP-β-CD lyophilized product, diminishes the extent of injury, having protective effect on the liver and kidneys after hepatic IRI.

The purpose of the present experimental protocol was to evaluate the possible protective effect of silibinin, when administered intravenously in the form of its SLB-HP-β-CD lyophilized product, on the observed acute lung injury and AKI after hepatic IRI. For this purpose, the expressions of MMP2, MMP3, MMP9 and TIMP2 on kidney and lung tissues were assessed.

## Methods

### Animals and reagents

Healthy male Wistar rats were utilized for the needs of the present study. All rats in the facility underwent regular screening according to a health-monitoring program, in compliance with the Federation of European Laboratory Animal Science Associations’ recommendations. The study was approved by the Veterinary Authorities of Region of Athens, Greece (583/05-02-2015). The animal experiments were performed at the Department of Experimental Surgery, Bioresearch Foundation of the Academy of Athens. The immunohistochemistry study was performed at the Laboratory of Histology-Embryology, Faculty of Medicine, Democritus University of Thrace. The real-time quantitative reverse transcription polymerase chain reaction (qRT-PCR) study was performed at the School of Health Sciences, Department of Pharmacy, National and Kapodistrian University of Athens.

Preparation of SLB-HP-β-CD lyophilized product was implemented as previously described^27^^,^ 44. Briefly, 0.300 g of silibinin (MW=482.44) and 1.860 g of HP-β-CD (MW=1,540) (both purchased from Sigma Aldrich, Steinheim, Germany, purity >99%) were transferred in a 300 mL volumetric flask and suspended with 200 mL of water (triple-deionized water from Millipore). Under continuous stirring and pH monitoring, small amounts of ammonium hydroxide were added until complete dissolution and pH adjustment between 9 and 10. The solution at a molar ratio of 1:2 was freeze-dried by the usage of a Biobase Vacuum Freeze Dryer, BK-FD10T, Biobase Biodustry (Shandong) Co., to remove water and produce the water soluble lyophilized SLB-HP-β-CD product. This powder was reconstituted in water for injection prior to administration, and the pH of the resulted solution was almost neutral.

### Experimental protocol

Sixty-three Wistar male rats were assigned in one of three different groups, namely sham, control and silibinin groups. The median age of the animals was 13 weeks old, and their average weight was 314 g. In the sham group (n=7 rats), there was no intervention apart from opening and closing the abdomen. In the control group (C; n=28 rats), a 45-min ischemia was applied followed by reperfusion. In the silibinin group (Si; n = 28 rats), ischemia was applied again for 45 min, and before reperfusion, SLB-HP-β-CD lyophilized product was administered intravenously.

The control and silibinin groups were then subdivided into time-point subgroups according to the duration of reperfusion and euthanasia time (i.e., C60, C120, C180, and C240 for 60, 120, 180 and 240 min, respectively, for the C subgroups, and Si60, Si120, Si180 and Si240, respectively, for the Si subgroups).

The surgical procedure included placing the animals in a supine position and administration of isoflurane for general anesthesia. Also, proper analgesia based on subcutaneous administration of carpofen (4 mg/kg) was maintained for all groups.

Amid line laparotomy was performed under sterile conditions. For the sham group, there was only an open-close laparotomy. For the C group, a 45-min Pringle maneuver was performed by placing a micro-clip around the hepatoduodenal ligament, that was removed afterwards. Prior to the removal of the micro-clip, 200 μL of water for injection was administered intravenously. For the Si group, a 45-min Pringle maneuver was similarly performed, and prior to the removal of the micro-clip, 200 μL of SLB-HP-β-CD lyophilized product, reconstituted in water for injection, was administered intravenously. The SLB concentration in the administered solution was 7.5 mg/mL. Therefore, 200 μL of this solution contained an administered dose of 1.5 mg of silibinin, and its selection was based on literature data^44^^,^ 46.

### Sample collection

Euthanasia was performed at the selected time points. Then, kidney and lung tissue samples were collected. Tissue samples from each group were snap-frozen in liquid nitrogen and stored at -80°C until usage. Tissue specimens were fixed in formalin 10% and were paraffin embedded according to routine histological practice.

### qRT-PCR protocol

#### RNA isolation and cDNA synthesis

Total RNA was isolated using the NucleoZOL reagent, according to the manufacturer’s instructions (Macherey-Nagel). Briefly, tissue specimens (~100 mg) were homogenized with the addition of 1 mL of NucleoZOL reagent. Contaminants were precipitated by adding 400 μL water/mL reagent, followed by rigorously mixing and incubation for 15 min at room temperature. After centrifugation for 15 min at 12,000 g, the supernatants were transferred into new tubes. Total RNAs were precipitated after addition of 1 mL of isopropanol, incubation for 10 min at room temperature and centrifugation at 12,000 g for 10 min at 4°C. The resulted RNA pellets were washed twice with 75% ethanol and centrifuged at 6,000 g for 3 min at 4°C. After ethanol removal by decantation, RNAs were reconstituted in nuclease-free H_2_O. RNA concentration and quality were accessed by spectrophotometry. All RNA samples were stored at -80°C.

First-strand cDNA was synthesized from 1 μg total RNA, using the Transcriptor First Strand cDNA Synthesis Kit (Roche Diagnostics, Mannheim, Germany), according to the manufacturer’s instructions. Each DNA-free RNA sample was added in a 20 μL reaction containing the appropriate volumes of cDNA synthesis buffer, random primers, RNAse inhibitor (40 u) deoxynucleotide mix and transcriptor reverse transcriptase and incubated at 25°C for 10 min, followed by incubation at 55°C for 30 min, and finally at 85°C for 5 min. All cDNA samples were stored at -20°C until further analysis.

#### qRT-PCR methodology

For qRT-PCR, gene-specific suitable pairs of primers and hybridization sets of dual probes (labelled with fluorescein donor and LC-Red 640 acceptor dyes) were used, as described in detail in Table 1. Each of the predictedqRT-PCR product spanned an intron–exon junction. Primers and probes were designed and synthesized by TIB Molbiol. Glyceraldehyde 3-phosphate dehydrogenase (GAPDH) was amplified as an internal control.

**Table 1:** Sequences of primers and probes for RT-PCR methodologies.

Target primer/ probe	Oligonucleotide sequence (5’→3’)
MMP-9 F	*GCTTTGCTGATGCTTCAGAA*
MMP-9 R	*CAGAGTAGTTTTGGATCCAGTATGTG*
MMP-9 FL	CGAATGGCCTTTAGTGTCTGGCT- FL
MMP-9 LC	*LC640 TCCAGCTCACCAGTCTGGGGCA PH*
TIMP-2 F	*CAGTATGAGATCAAGCAGATAAAGATGTT*
TIMP-2 R	*GCTCTTCTCTGTGACCCAGTC*
TIMP-2 FL	GTACCAGATGGGCTGTGAGTGCAAGA FL
TIMP-2 LC	LC CACTCGCTGTCCCATGATCCCTTG PH
MMP-2 F	*AGACAAAGAGTTGGCAGTGCAAT*
MMP-2 R	*CTGTATGTGATCTGGTTCTTGTCCC*
MMP-2 FL	TCCGCATGGTCTCGATGGTGTTCTG FL
MMP-2 LC	LC640 TCAAGGTCACCTGTCTGGGGCAGCC PH
MMP-3 F	*AGTGTGGATTCTGCCATTG*
MMP-3 R	*GAGTTCCATAGAGGGACTGAATAC*
MMP-3 FL	CGTTCATCATCGTCAAAGTGAGCATC FL
MMP-3 LC	LC640 CCATTAATCCCTGGTCCAGGTGCA PH
GAPDH F	*ATTCAACGGCACAGTCAAGG*
GAPDH R	*GCATTAGCTTCAGATTTACGG*
GAPDH FL	CCAGAAGACTGTGGATGGCCCCT FL

RT-PCR: reverse transcription polymerase chain reaction; MMP: matrix metalloproteinase; GAPDH: glyceraldehyde 3-phosphate dehydrogenase.

The qRT-PCR was performed using the LightCycler® FastStart DNA Master HybProbe kit, according to the manufacturer’s instructions (Roche Diagnostics, Mannheim, Germany) on the Light Cycler 2.0 real-time instrument (Roche Diagnostics, Mannheim, Germany) in glass capillaries in the total volume of 20 μL. For the transcripts, 2 μL of the sample cDNA was added to 1 μL of the forward primer (with final concentration of 0.5 μM), 1 μL of the reverse primer (with final concentration 0.5 μM), 1 μL of the sensor probe (with final concentration 0.2 μM), 1 μL of the anchor probe (with final concentration 0.2 μM), 1.8 μL MgCl_2_ (Roche Diagnostics, Mannheim, Germany, final concentration 2.25 mM), 2 μL Light Cycler Fast Start DNA Master HybProbe 10X (with final concentration 1X), and ddH_2_O to the final volume.

All reactions were performed according to the protocol described ahead. After pre-denaturation at 95°C for 10 min, the following was carried out for 50 cycles: denaturation at 95°C for 5 s, annealing at 60°C for 10 s, extension at 72°C for 5 s, and a cooling step at 40°C for 30 s in the last cycle. Relative expressions of MMP2, MMP3, MMP9 and TIMP2 transcript were performed by the usage of the comparative Ct method^48^, a mathematical model that calculates changes in gene expression as a relative fold difference between an experimental and calibrator sample.

More specifically by using the ΔΔCt method, the Relative quantity can be calculated by Equation 1:


$$R=2^{-\mathrm{\Delta \Delta Ct}}$$ (1)

In which:

ΔΔCt = ΔCt (Si group) - ΔCt (control group);

ΔCt = Ct (gene of interest) - Ct (GAPDH internal control).

R values greater than 1 reflect a positive difference in the gene expression of Si group compared to the control animals, i.e., an increase in the expression of the gene of interest, while a value of R <1 reflects a negative difference in the gene expression of Si group in comparison with the control animals, i.e., a decrease in the expression of the gene of interest.

### Immunohistochemistry protocol

Four-μM sections of representative blocks from each case were deparaffinized, rehydrated and treated with 0.3% H_2_O_2_ for 5 min in methanol in order to prevent endogenous peroxidase activity. Afterwards, sections were immunostained by the peroxidase method (Super Sensitive One-Step Polymer-HRP Detection System, QD 630-XAKE, Biogenex). Slides were then incubated for 60 min with the antibodies MMP2 (mouse monoclonal, Novus Biological, NB 200-114) at 1:300 dilution, MMP3 (rabbit polyclonal, Nobus Biologicals, NB100-91878) at 1:70 dilution, MMP9 (rabbit polyclonal, Novus Biologicals, NBP1-57940) at 1:250 dilution, and TIMP2 (mouse monoclonal, Novus Biologicals, NBP2-01573) at 1:300 dilution. Control slides were incubated for 60 min as well, with nonimmunized rabbit serum (negative control). A positive control was always run in the assay.

Bound antibody complexes, finally, were stained for 10 min with 0.05% diaminobenzidine. Afterwards, sections were briefly counterstained with Mayer’s haematoxylin, mounted and examined under a Nikon Eclipse 50i microscope. The positive expression of antibodies was assessed by counting the number of stained cells (cytoplasmic or nuclear localization). The average labeling antibodies index was determined according to the proportion of positive cells, after scanning the entire section of the specimen. The levels of expression were evaluated as negative (0) for <10% of stained cells, low (1) for >10% and <30% of stained cells, moderate (2) for >30% and <70% cells stained, and high (3) for >70% cells stained.

### Statistical analysis

Expression of MMP2, MMP3, MMP9 and TIMP2 (from IHC) are presented with absolute and relative frequencies and median values with interquartile range (IQR). qRT-PCR results, expressed as relative to internal control (GAPDH) differences (ΔCt), are presented with box and whiskers plots. Kolmogorov-Smirnov test revealed data were not normally distributed. Accordingly, the Kruskal-Wallis test was applied for the comparison of Si, C, and sham groups, while the Dunn’s test, with the respective Bonferroni adjustment of p-values for multiple testing, was used for post-hoc pairwise comparisons. Two-tailed p-values are reported. Statistical significance was considered at 0.05. Statistical analyses were performed using both the Statistical Package for the Social Sciences (SPSS) (version 26) and the R statistical package version 4.1.0.

## Results

### Immunohistochemistry

**Figure 1:**
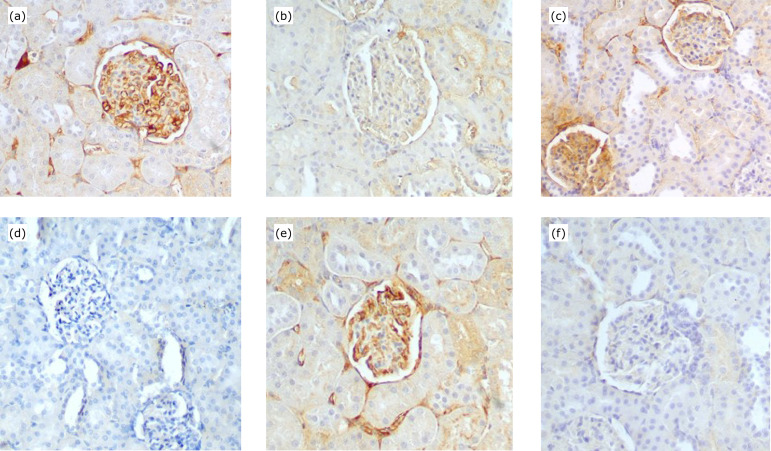
Photomicrographs of MMP2 (a, b), MMP3 (c, d) and MMP9 (e, f) in kidney of control (a,c,e) and silibinin (b,d,f) groups at 240 min (x200).

**Figure 2:**
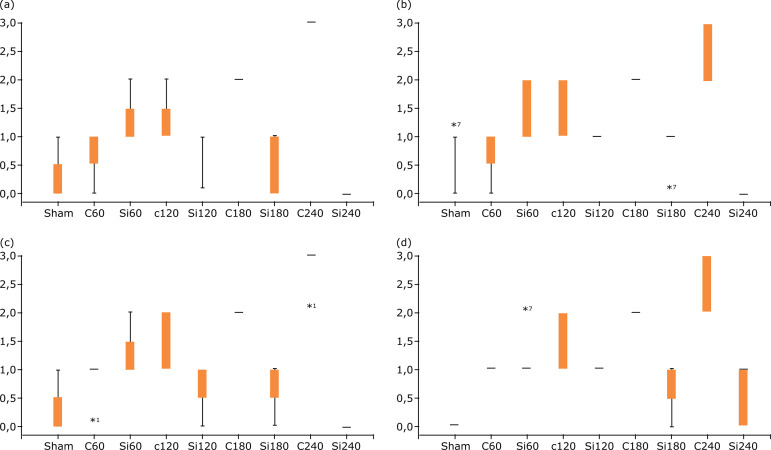
Expression of kidney: (a) MMP2; (b) MMP3; (c) MMP9; (d) TIMP2. Middle boxes represent the inter-quartile range. Whiskers represent the lower and upper scores. Stars indicate the presence of outliers in the data set. A number indicates the serial number of the animal which has the outlying score. Kruskal-Wallis test (Dunn’s test with Bonferroni adjustment).

The difference in the expression of MMP2 in kidney between C and Si groups was not significant at 120 and 180 min, but a significantly lower expression was found for the Si group at 240 min (Fig. 1a,b; Fig. 2a; Table 2). Silibinin group at 240 min had significantly lower expression of MMP3 in kidney, as compared to respective C group (Fig. 1c, d; Fig. 2b; Table 2). The difference in the expression of MMP9 in kidney between C and Si group did not reach statistical significance at 60, 120 and 180 min, but a significantly lower expression was found for the Si group at 240 min (Fig. 1e,f; Fig. 2c; Table 3). As shown in Table 3, the expression of TIMP2 in kidney was lower at 180 and 240 min (Fig. 2d).

**Table 2:** Expression of MMP2 and MMP3 in kidney for all study groups and the results of the statistical analysis * .

	MMP2 kidney						MMP3 kidney				
	0	1	2	3	Median		0	1	2	3	Median
Group	N (%)	N (%)	N (%)	N (%)	(25th-75th)		N (%)	N (%)	N (%)	N (%)	(25th-75th)
Sham	5 (71.4)	2 (28.6)	0 (0.0)	0 (0.0)	0 (0-1)		6 (85.7)	1 (14.3)	0 (0.0)	0 (0.0)	0 (0-0)
C60	2 (28.6)	5 (71.4)	0 (0.0)	0 (0.0)	1 (0-1)		2 (28.6)	5 (71.4)	0 (0.0)	0 (0.0)	1 (0-1)
C120	0 (0.0)	5 (71.4)	2 (28.6)	0 (0.0)	1 (1-2)		0 (0.0)	4 (57.1)	3 (42.9)	0 (0.0)	1 (1-2)
C180	0 (0.0)	0 (0.0)	7 (100.0)	0 (0.0)	2 (2-2)		0 (0.0)	0 (0.0)	7 (100.0)	0 (0.0)	2 (2-2)
C240	0 (0.0)	0 (0.0)	0 (0.0)	7 (100.0)	3 (3-3)		0 (0.0)	0 (0.0)	3 (42.9)	4 (57.1)	3 (2-3)
Si60	0 (0.0)	5 (71.4)	2 (28.6)	0 (0.0)	1 (1-2)		0 (0.0)	4 (57.1)	3 (42.9)	0 (0.0)	1 (1-2)
Si120	0 (0.0)	7 (100.0)	0 (0.0)	0 (0.0)	1 (1-1)		0 (0.0)	7 (100.0)	0 (0.0)	0 (0.0)	0 (0-0)
Si180	3 (42.9)	4 (57.1)	0 (0.0)	0 (0.0)	1 (0-1)		1 (14.3)	6 (85.7)	0 (0.0)	0 (0.0)	1 (1-1)
Si240	7 (100.0)	0 (0.0)	0 (0.0)	0 (0.0)	0 (0-0)		7 (100.0)	0 (0.0)	0 (0.0)	0 (0.0)	0 (0-0)
P Sham vs. C60					1.000						1.000
P Sham vs. C120					0.682						0.179
P Sham vs. C180					0.004						0.003
P Sham vs. C240					<0.001						<0.001
P C60 vs. Si60					1.000						1.000
P C120 vs. Si120					1.000						0.061
P C180 vs. Si180					0.057						0.850
P C240 vs. Si240					<0.001						<0.001

N: the number of animals and in parentheses the inter-quartile range; 25^th^-75^th^: the 25^th^-75^th^ percentile; P: p-value from the Kruskal-Wallis test;

* the nonparametric Dunn’s test was also applied for the comparison of Si, C and sham groups with the respective Bonferroni adjustment of p-values.

**Table 3:** Expression of MMP9 and TIMP2 in kidney for all study groups and the results of the statistical analysis * .

	MMP9 kidney						TIM2 kidney				
	0	1	2	3	Median		0	1	2	3	Median
Group	N (%)	N (%)	N (%)	N (%)	(25th-75th)		N (%)	N (%)	N (%)	N (%)	(25th-75th)
Sham	5 (71.4)	2 (28.6)	0 (0.0)	0 (0.0)	0 (0-1)		7 (100.0)	0 (0.0)	0 (0.0)	0 (0.0)	0 (0-0)
C60	1 (14.3)	6 (85.7)	0 (0.0)	0 (0.0)	1 (1-1)		0 (0.0)	7 (100.0)	0 (0.0)	0 (0.0)	1 (1-1)
C120	0 (0.0)	4 (57.1)	3 (42.9)	0 (0.0)	1 (1-2)		0 (0.0)	3 (42.9)	4 (57.1)	0 (0.0)	2 (1-2)
C180	0 (0.0)	0 (0.0)	7 (100.0)	0 (0.0)	2 (2-2)		0 (0.0)	0 (0.0)	7 (100.0)	0 (0.0)	2 (2-2)
C240	0 (0.0)	0 (0.0)	1 (14.3)	6 (85.7)	3 (3-3)		0 (0.0)	0 (0.0)	4 (57.1)	3 (42.9)	2 (2-3)
Si60	0 (0.0)	5 (71.4)	2 (28.6)	0 (0.0)	1 (1-2)		0 (0.0)	6 (85.7)	1 (14.3)	0 (0.0)	1 (1-1)
Si120	2 (28.6)	5 (71.4)	0 (0.0)	0 (0.0)	1 (0-1)		0 (0.0)	7 (100.0)	0 (0.0)	0 (0.0)	1 (1-1)
Si180	2 (28.6)	5 (71.4)	0 (0.0)	0 (0.0)	1 (0-1)		2 (28.6)	5 (71.4)	0 (0.0)	0 (0.0)	1 (0-1)
Si240	7 (100.0)	0 (0.0)	0 (0.0)	0 (0.0)	0 (0-0)		4 (57.1)	3 (42.9)	0 (0.0)	0 (0.0)	0 (0-1)
P Sham vs. C60					1.000						0.548
P Sham vs. C120					0.179						0.003
P Sham vs. C180					0.003						<0.001
P Sham vs. C240					<0.001						<0.001
P C60 vs. Si60					1.000						1.000
P C120 vs. Si120					0.061						1.000
P C180 vs. Si180					0.850						0.037
P C240 vs. Si240					<0.001						<0.001

N: the number of animals and in parentheses the inter-quartile range; 25^th^-75^th^: the 25^th^-75^th^ percentile; P: the p-value from the Kruskal-Wallis test;

* the nonparametric Dunn’s test was also applied for the comparison of Si, C and sham groups with the respective Bonferroni adjustment of p-values.

**Figure 3:**
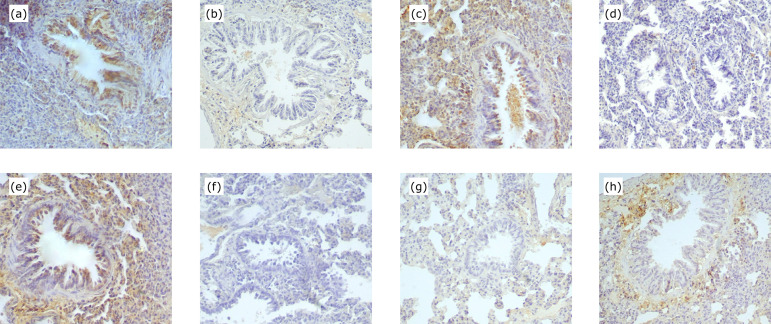
Photomicrographs of MMP2 **(a, b)**, MMP3 **(c, d)**, MMP9 **(e, f)** and TIMP2 **(g, h)** in lung of control **(a, c, e, g)** and silibinin **(b, d, f, h)** groups at 240 min for MMP2, MMP3 and MMP9 and 180 min for TIMP2 (x200).

**Figure 4:**
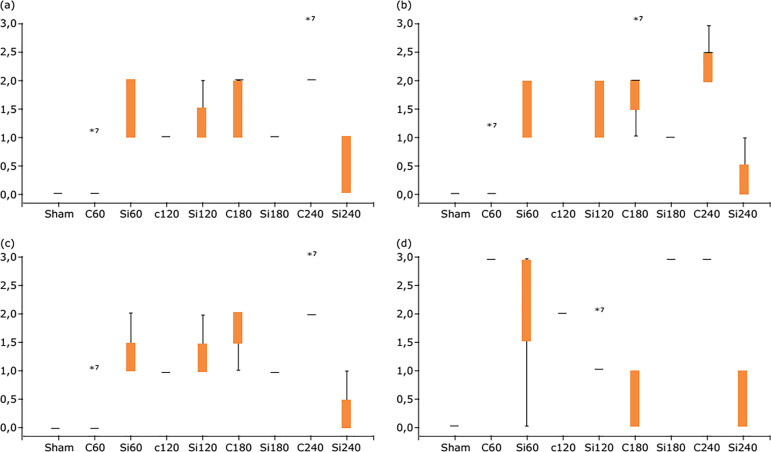
Expression of lung: **(a)**MMP2;**(b)**MMP3;**(c)**MMP9;**(d)**TIMP2. Middle boxes represent the inter-quartile range. Whiskers represent the lower and upper scores. Stars indicate the presence of outliers in the data set. A number indicates the serial number of the animal which has the outlying score. Kruskal-Wallis test (Dunn’s test with Bonferroni adjustment).

The difference in the expression of MMP2 in lung between C and Si groups was not significant at 120 and 180 min, but a significantly lower expression was recorded for the Si group at 240 min. When comparing C60–Si60 groups, MMP2 values in lung were statistically significantly higher for the Si group (Fig. 3 a,b; Fig. 4a; Table 4). Expression of MMP3 in lung is presented in Table 4. The expression of MMP3 in lung in the Si groups was significantly lower compared to the C group at 180 and 240 min (Fig. 3c,b; Fig. 4b; Table 4). The difference in the expression of MMP9 in lung between C and Si groups was not significant at 120 and 180 min, but a significantly lower expression was found for the Si group at 240 min (Fig. 3e,f; Table 5). Silibinin groups at 60, 120 and 240 min did not have lower expression of TIMP2 in lung as compared to C groups at these time points. When comparing C180-Si180 groups, TIMP2 values were statistically significantly lower for the C group (Fig. 3g,h; Fig. 4d; Table 5).

**Table 4:** Expression of MMP2 and MMP3 in lung for all study groups and the results of the statistical analysis * .

	MMP2 lung					MMP3 lung				
	0	1	2	3	Median	0	1	2	3	Median
Group	N (%)	N (%)	N (%)	N (%)	(25th-75th)	N (%)	N (%)	N (%)	N (%)	(25th-75th)
Sham	7 (100.0)	0 (0.0)	0 (0.0)	0 (0.0)	0 (0-0)	7 (100.0)	0 (0.0)	0 (0.0)	0 (0.0)	0 (0-0)
C60	6 (85.7)	1 (14.3)	0 (0.0)	0 (0.0)	0 (0-0)	6 (85.7)	1 (14.3)	0 (0.0)	0 (0.0)	0 (0-0)
C120	0 (0.0)	7 (100.0)	0 (0.0)	0 (0.0)	1 (1-1)	0 (0.0)	7 (100.0)	0 (0.0)	0 (0.0)	1 (1-1)
C180	0 (0.0)	3 (42.9)	4 (57.1)	0 (0.0)	2 (1-2)	0 (0.0)	2 (28.6)	4 (57.1)	1 (14.3)	2 (1-2)
C240	0 (0.0)	0 (0.0)	6 (85.7)	1 (14.3)	2 (2-2)	0 (0.0)	0 (0.0)	5 (71.4)	2 (28.6)	2 (2-3)
Si60	0 (0.0)	4 (57.1)	3 (42.9)	0 (0.0)	1 (1-2)	0 (0.0)	4 (57.1)	3 (42.9)	0 (0.0)	1 (1-2)
Si120	0 (0.0)	5 (71.4)	2 (28.6)	0 (0.0)	1 (1-2)	0 (0.0)	3 (42.9)	4 (57.1)	0 (0.0)	2 (1-2)
Si180	0 (0.0)	7 (100.0)	0 (0.0)	0 (0.0)	1 (1-1)	0 (0.0)	7 (100.0)	0 (0.0)	0 (0.0)	1 (1-1)
Si240	4 (57.1)	3 (42.9)	0 (0.0)	0 (0.0)	0 (0-1)	5 (71.4)	2 (28.6)	0 (0.0)	0 (0.0)	0 (0-1)
P Sham vs. C60					1.000					1.000
P Sham vs. C120					0.348					0.623
P Sham vs. C180					0.002					0.001
P Sham vs. C240					<0.001					<0.001
P C60 vs. Si60					0.037					0.093
P C120 vs. Si120					1.000					1.000
P C180 vs. Si180					1.000					1.000
P C240 vs. Si240					0.002					0.001

N: the number of animals and in parentheses the inter-quartile range; 25^th^-75^th^: the 25^th^-75^th^ percentile; P: the p-value from the Kruskal-Wallis test;

* the nonparametric Dunn’s test was also applied for the comparison of Si, C and sham groups with the respective Bonferroni adjustment of p-values.

**Table 5:** Expression of MMP9 and TIMP2 in lung for all study groups and the results of the statistical analysis * .

	MMP9 lung					TIM2 lung				
	0	1	2	3	Median	0	1	2	3	Median
Group	N (%)	N (%)	N (%)	N (%)	(25th-75th)	N (%)	N (%)	N (%)	N (%)	(25th-75th)
Sham	7 (100.0)	0 (0.0)	0 (0.0)	0 (0.0)	0 (0-0)	7 (100.0)	0 (0.0)	0 (0.0)	0 (0.0)	0 (0-0)
C60	6 (85.7)	1 (14.3)	0 (0.0)	0 (0.0)	0 (0-0)	0 (0.0)	0 (0.0)	0 (0.0)	7 (100.0)	3 (3-3)
C120	0 (0.0)	7 (100.0)	0 (0.0)	0 (0.0)	1 (1-1)	0 (0.0)	0 (0.0)	7 (100.0)	0 (0.0)	2 (2-2)
C180	0 (0.0)	2 (28.6)	5 (71.4)	0 (0.0)	2 (1-2)	4 (57.1)	3 (42.9)	0 (0.0)	0 (0.0)	0 (0-1)
C240	0 (0.0)	0 (0.0)	6 (85.7)	1 (14.3)	2 (2-2)	0 (0.0)	0 (0.0)	0 (0.0)	7 (100.0)	3 (3-3)
Si60	0 (0.0)	5 (71.4)	2 (28.6)	0 (0.0)	1 (1-2)	2 (28.6)	0 (0.0)	0 (0.0)	5 (71.4)	3 (0-3)
Si120	0 (0.0)	5 (71.4)	2 (28.6)	0 (0.0)	1 (1-2)	0 (0.0)	6 (85.7)	1 (14.3)	0 (0.0)	1 (1-1)
Si180	0 (0.0)	7 (100.0)	0 (0.0)	0 (0.0)	1 (1-1)	0 (0.0)	0 (0.0)	0 (0.0)	7 (100.0)	3 (3-3)
Si240	5 (71.4)	2 (28.6)	0 (0.0)	0 (0.0)	0 (0-1)	4 (57.1)	3 (42.9)	0 (0.0)	0 (0.0)	0 (0-3)
P Sham vs. C60					1.000					<0.001
P Sham vs. C120					0.358					0.691
P Sham vs. C180					0.001					1.000
P Sham vs. C240					<0.001					<0.001
P C60 vs. Si60					0.135					1.000
P C120 vs. Si120					1.000					1.000
P C180 vs. Si180					1.000					0.006
P C240 vs. Si240					<0.001					0.460

N: the number of animals and in parentheses the inter-quartile range; 25^th^-75^th^: the 25^th^-75^th^ percentile; P: the p-value from the Kruskal-Wallis test;

* the nonparametric Dunn’s test was also applied for the comparison of Si, C and Sham groups with the respective Bonferroni adjustment of p-values.

### qRT-PCR

**Figure 5:**
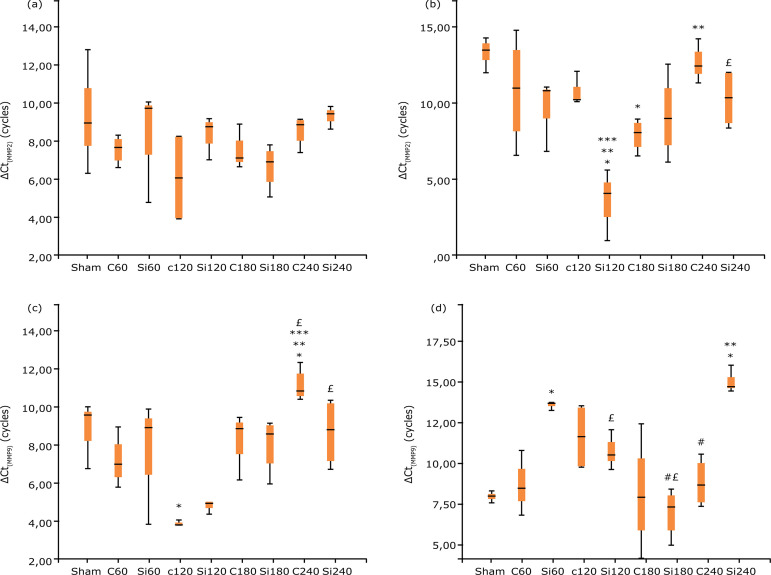
Box and Whiskers plot for gene expression of MMPs in kidneys after liver IRI in Wistar rats: ΔCt_MMP2_ **(a)**, ΔCt_MMP3_ **(b)**, ΔCt_MMP9_ **(c)** and ΔCt_TIMP2_ **(d)**. Kruskal-Wallis test (Dunn’s test with Bonferroni adjustment). Statistically significant difference p<0.05:

**Figure 6:**
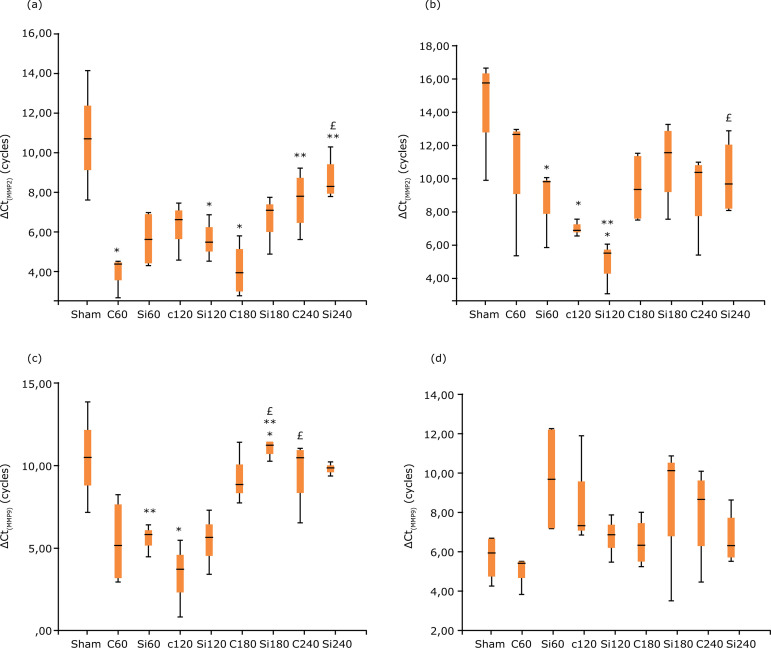
Box and Whiskers plot for gene expression of MMPs in lungs after liver IRI in Wistar rats: ΔCt_MMP2_ **(a)**, ΔCt_MMP3_ **(b)**, ΔCt_MMP9_ **(c)** and ΔCt_TIMP2_ **(d)**. Kruskal-Wallis test (Dunn’s test with Bonferroni adjustment). Statistically significant difference p<0.05.

Results of qRT-PCR are shown in Figures 5 and 6. As far as kidney MMP2 is concerned, despite the trend for decreased gene expression in the Si group, due to the high variability, there is no statistically significant difference between C and Si groups at the same time points (Fig. 5a). Animals of Si group have a significantly lower expression of kidney MMP3 at 240 min and higher ΔCt value compared to the C group animals (Fig. 5b). Furthermore, expression values of kidney MMP9 are significantly lower for the Si group at 180 and 240 min (and higher ΔCt values), when compared to the C at the same time points (Fig. 5c). TIMP2 expression values of renal parenchyma are significantly higher for the Si group at 120 min (and lower ΔCt value), while they decrease to a statistically significant level at 240 min (and higher ΔCt value), when compared to the values of the C group (Fig. 5d).

*In comparison to sham group, sham; **from time point 60 min of the same group; ***from time point 120 min of the same group; # from time point 240 min of the same group; £ between control group, C and Silibinin group, Si, at the same time point; MMPs: matrix metalloproteinases; I/R: ischemia/reperfusion injury.

*In comparison to sham group, sham; **from time point 60 min of the same group; ***from time point 120 min of the same group; # from time point 240 min of the same group; £ between control group, C and Silibinin group, Si, at the same time point; MMPs: matrix metalloproteinases; I/R: ischemia/reperfusion injury.

Lung MMP2 and MMP3 expressions, as shown by the qRT-PCR method, are statistically significantly lower for the Si group at 240 min, when compared to the C group (Fig. 6 a,b), while MMP9 has significantly lower values for the animals of the Si group at all time points compared to the animals of the C group at the same time points (Fig. 6 b,c). As far as TIMP2 expression in lung parenchyma is concerned, there is no statistically significant difference between the two groups (Fig. 6d), probably due to the high variability observed.

These results are also reflected on the calculated Relative quantity (R=2^–ΔΔCt^) values, presented in Table 6.

**Table 6:** Relative quantity (R=2^-ΔΔCt^) values for the effect of silibinin on MMP2, MMP3, MMP9 and TIMP2 gene expression in kidney and lung after hepatic IRI.

Time after I/R (min)	Kidney			
	R=2^-ΔΔCt^			
	MMP-2	MMP-3	MMP-9	TIMP-2
60	1.04	7.77	0.443	1.46
120	2.87	1.33	0.701	102
180	0.18	0.28	0.006	28.1
240	0.52	0.009	0.065	0.049
**Time after I/R (min)**	**Lung**			
	**R=2^-ΔΔCt^**			
	**MMP-2**	**MMP-3**	**MMP-9**	**TIMP-2**
60	0.87	2.81	0.075	0.345
120	0.48	0.18	0.023	2.11
180	0.38	0.211	0.013	1.01
240	0.12	0.025	0.050	1.07

I/R: ischemia/reperfusion injury.

## Discussion

In the present study, distal renal and lung injury caused by hepatic IRI and the time-dependent protective action of silibinin were studied. AKI as well as ALI following hepatic IRI were confirmed by the elevated expression levels of MMP2, MMP3, MMP9 and TIMP2 in the control groups compared to sham.

The protective effect of silibinin on renal parenchyma was reflected on the observed reduced expression of MMP2 in the Si group compared to the C group. Also, its protective effect was confirmed by the reduced levels of expression of MMP3, MMP9 and TIMP2 in the Si group animals, as compared to the animals of the C group, as shown by the results of immunohistochemistry method. The protective effect of silibinin on rat kidney was also supported by qRT-PCR results, showing reduction in the expression of kidney MMP3, for the animals of Si group, compared to the C group animals. The silibinin renoprotective effect was also supported by the statistically significantly reduced expression level of kidney MMP9 and by the increased expression level of kidney TIMP2.

The protective effect of Silibinin on lung parenchyma was reflected on the observed reduced expressions of MMP2, MMP3 and MMP9 in the Si group compared to the C group. At 180 min after reperfusion, the level of expression of TIMP2 was notably elevated for the animals of the Si group. This makes a shift in the balance of MMP–TIMP2 in favor of TIMP2, reflecting the high protective effect of Si administration on lung parenchyma at that time point.

IHC findings reflecting Silibinin protective action on lung parenchyma are supported by qRT-PCR results, showing reduced expression levels of lung MMP2 and MMP3 at 240 min. Its protective effect was also demonstrated by the statistically significantly decreased expression level of lung MMP9 at all time points after IRI for the animals of the Si group compared to those of the C group.

Hepatic IRI causes significant ischemic injury in the hepatic parenchyma. Aside from that, it causes damage in remote organs such as kidneys and lungs through the production of pro-inflammatory mediators like TNF-a, IL-6, IL-1, and oxygen free radicals^49^^-^ 52. AKI and ALI following hepatic IRI are assessed by the level of expression of MMP2, MMP3, MMP9 and one of their inhibitors, TIMP2, a family of zinc-dependent endopeptidases that degrade extracellular matrix^31^ and are involved in many kidney and lung diseases^32^^-^ 37.

Previous studies have shown that hepatic IRI is strongly associated with AKI^14^^,^ 45 ^,^ 53. As shown in the study by Lee *et al*.^14^, rats that underwent IRI for 60 min developed significant acute renal failure within 24 hours. Similarly, in Polat *et al*.^15^, renal functions were disturbed, and the level of oxidative stress was increased after 45 min of hepatic ischemia followed by 60 min of reperfusion in an experimental rat model. Kyriakopoulos *et al*.^45^ observed significant renal damage after 45 min of hepatic ischemia followed by 120 min of reperfusion in a rat model. In the present study, significant renal damage was recorded after 45 min of ischemia followed by 180 and 240 min of reperfusion.

Abu-Amara *et al*.^54^ tested the protective effect of nitric oxide, which maintains sufficient blood flow in the microcirculation of target organ, against the action of pro-inflammatory mediators released by IRI. Ramalho *et al*.^55^ studied the protective effect of rosmarinic acid on hepatocytes in a rat model of 60 min ischemia followed by 6 hours of reperfusion, concluding that rosmarinic acid reduced hepatocellular damage and all oxidative stress parameters. Sherif *et al*.^56^ found that vildagliptin ameliorated the remote renal injury that occurred after hepatic IRI by reducing the oxidative stress and the pro-inflammatory cytokine TNF-a. Meng *et al*.^57^ tested the renoprotective effect of polydatin against IRI by decreasing apoptosis and oxidative stress.

Sun *et al*.^58^ observed that octreotide reduced inflammation and apoptosis of renal tissue and preserved renal function by reducing the severity of injury in a rat model of 60 min hepatic ischemia followed by 24 hours of reperfusion. Abdel-Daim *et al*.^59^ tested the protective effect of rosuvastatin and vitamin E on liver and kidney against the damage caused by fipronil (FPN) in rats, concluding that they ameliorated the FPN induced hepatorenal toxicity through their anti-oxidative properties. In our study, the administration of silibinin after 45 min of hepatic ischemia showed its renoprotective effects at 180 and 240 min after reperfusion.

In previous studies, hepatic IRI inducing ALI has been well documented^12^^,^ 23 ^,^ 27 ^,^ 60 ^-^ 62. Ge *et al*.^60^ showed that hepatic IRI can induce remote ALI, accompanied by a significant increase of oxidative stress, in an experimental rat model of 60 min hepatic ischemia, reaching a pick at 6 hours after reperfusion, including alveolar damage and perivascular and peribronchial edema. In Colletti *et al*.^27^, hepatic IRI induced ALI is confirmed by a number of alterations in lung pathophysiology in a rat model of 90 min hepatic ischemia. In Chan *et al*.^23^, it is shown that hepatic IRI induced a significant deterioration of lung functions including edema formation in a rat model of 90 min ischemia followed by 5 hours of reperfusion. In the present study, significant lung injury was recorded after 45 min of liver ischemia followed by 180 and 240 min of reperfusion.

In the study of Chan *et al*.^23^, the protective effect of propofol on lung tissue was tested. It attenuated remote pulmonary effects by decreasing ROS production after hepatic IRI. Yu *et al*.^63^ tested the protective action of saquinavir on lung tissue, in an experimental rat model of 60 min ischemia followed by 6 and 12 hours of reperfusion, respectively. They demonstrated that administration of saquinavir attenuated lung injury by improving lung tissue and by reducing the expression of pro-inflammatory factors^63^. In our study, the protective effect of silibinin administration on lung parenchyma, after 45 min of hepatic ischemia, was shown at 180 and 240 min after reperfusion reaching a pick at 180 min.

Silibinin, a non-toxic polyphenolic flavonoid, is the main active component extracted from a medicinal plant named *Silybum marianum* 64. It was previously reported that silibinin has anti-inflammation, antifungal, antioxidant and antitumor activities^38^^-^ 44 ^,^ 46 ^,^ 47 ^,^ 65 ^-^ 67. Recently, experimental research protocols have been developed to study the possible protective activity of silibinin against IRI^46^^,^ 53 ^,^ 65.

In the present study, comparison between sham and C groups revealed that expression of MMP2, MMP3, MMP9 and TIMP2 in kidney was higher in the animals of the C group at all time points, providing supporting evidence of renal damage due to the hepatic IRI that started being statistically significant after 180 min of reperfusion. Comparing the expression of MMP2, MMP3, MMP9 and TIMP2 in lung, between sham and C groups, it was shown that the values of expression were significantly higher for the animals of the C group at 180 and 240 min, revealing that lung damage due to hepatic IRI becomes statistically significant after 120 min of reperfusion.

Regarding the protective action of silibinin on kidney parenchyma, it was observed that the expression of the studied genes was decreased in the animals of the Si group in a time dependent manner. More specifically, expression of MMP2, MMP3, and MMP9 was significantly lower for the Si group at 240 min. As far as the protective effect of silibinin on lung parenchyma is concerned, expression of MMP2, MMP3 and MMP9 was also significantly lower for the Si group at 240 min. Silibinin offers high protective action on lung parenchyma at 180 min after reperfusion timepoint when the expression of TIMP2 was notably elevated.

From the mentioned observations, it becomes clear the time-dependent protective effect of silibinin both on kidney and lung parenchyma, when administered intravenously after 45 min of hepatic ischemia and before reperfusion. All studied renal and lung parameters were significantly improved at time points of 240 min after reperfusion.

Regarding the limitations of the present study, it should be pointed that the presented results are limited to the single dose administration protocol followed. However, these results consist of great evidence and challenge to extent the studies and explore the possible dose dependent protective effect of silibinin in kidney and lung after hepatic IRI. Furthermore, additional studies demonstrating the activation of MMPs would further clarify the role of these proteins in hepatic IRI.

## Conclusion

Silibinin intravenous administration in the form of SLB-HP-β-CD lyophilized product presented time-dependent protective effect on kidney and lung parenchyma following hepatic IRI based on both immunohistochemistry and qRT-PCR.
